# Temporal and Potential Predictive Relationships between Sleep Spindle Density and Spike-and-Wave Discharges

**DOI:** 10.1523/ENEURO.0058-24.2024

**Published:** 2024-09-18

**Authors:** Manal S. Abdelaal, Tomonobu Kato, Akiyo Natsubori, Kenji F. Tanaka

**Affiliations:** ^1^Division of Brain Sciences, Institute for Advanced Medical Research, Keio University School of Medicine, Shinjuku-ku, Tokyo 160-8582, Japan; ^2^Department of System Design Engineering, Faculty of Science and Technology, Keio University, Kohoku-ku, Yokohama, Kanagawa 223-8522, Japan; ^3^Sleep Disorders Project, Tokyo Metropolitan Institute of Medical Science, Setagaya-Ku, Tokyo 156-8506, Japan

**Keywords:** absence seizures, cortico–thalamo–cortical network, electroencephalographic, sleep, sleep spindles, spike-and-wave discharges, tTA-tetO system

## Abstract

Spike-and-wave discharges (SWDs) and sleep spindles are characteristic electroencephalographic (EEG) hallmarks of absence seizures and nonrapid eye movement sleep, respectively. They are commonly generated by the cortico–thalamo–cortical network including the thalamic reticular nucleus (TRN). It has been reported that SWD development is accompanied by a decrease in sleep spindle density in absence seizure patients and animal models. However, whether the decrease in sleep spindle density precedes, coincides with, or follows, the SWD development remains unknown. To clarify this, we exploited *Pvalb*-tetracycline transactivator (tTA)::tetO-ArchT (PV-ArchT) double-transgenic mouse, which can induce an absence seizure phenotype in a time-controllable manner by expressing ArchT in PV neurons of the TRN. In these mice, EEG recordings demonstrated that a decrease in sleep spindle density occurred 1 week before the onset of typical SWDs, with the expression of ArchT. To confirm such temporal relationship observed in these genetic model mice, we used a gamma-butyrolactone (GBL) pharmacological model of SWDs. Prior to GBL administration, we administered caffeine to wild-type mice for 3 consecutive days to induce a decrease in sleep spindle density. We then administered low-dose GBL, which cannot induce SWDs in normally conditioned mice but led to the occurrence of SWDs in caffeine-conditioned mice. These findings indicate a temporal relationship in which the decrease in sleep spindle density consistently precedes SWD development. Furthermore, the decrease in sleep spindle activity may have a role in facilitating the development of SWDs. Our findings suggest that sleep spindle reductions could serve as early indicators of seizure susceptibility.

## Significance Statement

This study uncovers a crucial temporal link between sleep spindle reduction and the onset of spike-and-wave discharges (SWDs), hallmarks of absence seizures. By demonstrating that a decrease in sleep spindle density precedes SWD development in both genetic and pharmacological mouse models, our findings suggest that sleep spindle alterations could serve as early indicators of seizure susceptibility. This research opens new avenues for early detection and intervention strategies in absence seizures, potentially improving patient outcomes and advancing our understanding of seizure mechanisms.

## Introduction

The relationship between spike-and-wave discharges (SWDs), an electroencephalographic (EEG) hallmark of absence seizures, and sleep spindles, a prominent characteristic of nonrapid eye movement (NREM) sleep, has been a topic of interest in the field of epilepsy. Several studies have investigated the structural relationship between these two types of oscillations (Gloor, 1968; Kostopoulos et al., 1981; Sitnikova, 2010; Kozák et al., 2020). These oscillations are believed to originate from the same cortico–thalamo–cortical (CTC) networks, which include the corticothalamic and thalamocortical circuits and the thalamic reticular nucleus (TRN) (Steriade et al., 1993; Avoli, 2012; Lüthi, 2014; Fernandez and Lüthi, 2020). Previous studies have indicated a link between SWD development and alteration in sleep spindle patterns. Specifically, children with SWDs exhibited lower sleep spindle density during NREM sleep compared with healthy children (Zhang et al., 2023). Similar findings have been observed in Wistar Albino Glaxo Rijswijk (WAG/Rij) rats, a validated genetic animal model of absence seizures, and in some Long–Evans rats (Akman et al., 2010; Sitnikova et al., 2014; Kozák et al., 2020; Sitnikova, 2021). Studies on these rats have highlighted an age-dependent increase in epileptic activity alongside changes in the intrinsic dynamics of sleep spindle patterns. These investigations suggested that the occurrence of SWDs is associated with a reduction in sleep spindle density. However, the temporal relationship—whether the decrease in sleep spindle density precedes, coincides with, or follows the onset of SWDs—remains unclear.

This study aimed to investigate this temporal relationship between the development of SWDs and sleep spindle density. Specifically, it aimed to determine whether a decrease in sleep spindle density precedes or follows the onset of SWDs. To achieve this, a double-transgenic mouse [*Pvalb*-tetracycline transactivator (tTA)::tetO-ArchT] was used, which selectively expresses ArchT, an inhibitory opsin, in parvalbumin-positive (PV) cells in the TRN. The expression of ArchT in TRN-PV is sufficient to induce spontaneous, cortical 7–11 Hz SWDs (Abdelaal et al., 2022). Previous findings indicated that the PV-ArchT mice fulfilled the face and pharmacological validities of the two existing genetic rat models of absence seizures: WAG/Rij and Genetic Absence Epilepsy Rat from Strasbourg (GAERS) rats (Akman et al., 2010; Kandratavicius et al., 2014; Abdelaal et al., 2022). A unique aspect of the PV-ArchT model is its ability to consistently and controllably induce SWDs through the regulated expression of ArchT, dependent on the presence or absence of doxycycline (DOX) in the diet. In this study, a DOX-controllable approach was employed in PV-ArchT mice to investigate the temporal relationship between the development of SWDs and alterations in sleep spindle patterns.

SWDs in rat models have a similar pharmacological profile as humans. Drugs that enhance GABAergic inhibition have been found to exacerbate SWDs and decrease sleep spindles, while drugs that promote sleep spindles have been shown to decrease SWDs (Hirshkowitz et al., 1982; Van Luijtelaar, 1997; Sitnikova and van Luijtelaar, 2005). However, the link between reduced sleep spindle and the likelihood of SWD development is not yet fully understood. Therefore, a pharmacological mouse model was used to confirm the temporal relationship between sleep spindle alteration and SWD development and to explore the role of sleep spindle reduction on SWD development. We observed that administering a low dose (50 mg/kg) of gamma-butyrolactone (GBL), which is known to be insufficient to induce SWDs on its own, induced SWDs in wild-type mice whose sleep spindle density had been reduced by repeated prior caffeine treatment (Ishige et al., 1996; Venzi et al., 2015). These confirm the temporal relationship that sleep spindle reduction plays a predictive role in the development of SWDs. Also, sleep spindle reduction may have a role in facilitating the development of SWDs. Our findings suggest that sleep spindle alterations could serve as early indicators of absence seizures.

## Materials and Methods

### Ethics statement

All animal procedures were conducted according to the National Institutes of Health Guide for the Care and Use of Laboratory Animals and approved by the Animal Research Committee of Keio University School of Medicine.

### Experimental design

Male and female PV-ArchT double-transgenic mice were generated by crossing *Pvalb*-tTA mice with tetO-ArchT-EGFP mice aged 50–60 d (Sasaki et al., 2012; Tsunematsu et al., 2013; Abdelaal et al., 2022). For pharmacological experiments, C57BL/6J mice were employed. All mice were kept under a 12 h/12 h light/dark cycle (lights on at 8:00 A.M., lights off at 8:00 P.M.) in their home cages.

### Stereotaxic surgery

EEG/electromyography (EMG) electrode implantation was performed as described in a previous study (Abdelaal et al., 2022). For anesthesia, mice received a mixture of ketamine and xylazine (100 and 10 mg/kg, respectively) intraperitoneally. The cortical EEG electrodes were located above the parietal area (anteroposterior −1.5 mm, mediolateral +2.0 mm from the bregma). For EMG recording, silver wires (AS633; Cooner Wire) were inserted bilaterally into the trapezius muscles. The mice were allowed to recover for 1 week before recording.

### Pharmacological intervention

Ethosuximide (Tokyo Chemical Industry) was dissolved in normal saline and injected intraperitoneally into mice at a dose of 100 mg/kg. GBL (Sigma-Aldrich) was mixed with normal saline and injected intraperitoneally into mice at 50 and 100 mg/kg. Caffeine (Sigma-Aldrich) was dissolved in normal saline and injected intraperitoneally into mice at 12 mg/kg.

### DOX administration

DOX (Sigma-Aldrich) was administered to mice using chow (CE-2, CLEA) containing 100 mg/kg DOX as previously used in a DOX-dependent gene induction system (Tsutsui-Kimura et al., 2017; Abdelaal et al., 2022; Kato et al., 2023).

### EEG/EMG recordings

EEG/EMG recordings were performed on *ad libitum* moving mice in their home cages placed in a soundproof box. During the DOX-off or pharmacological experimental period, mice were repeatedly connected to EEG/EMG cables and monitored in their home cages for >24 h at a time, including both 12 h light and dark phases. EEG/EMG recordings were consistently synchronized with video monitoring. To acclimate mice to the recording procedures, they underwent three separate habituation sessions for EEG/EMG recordings prior to the commencement of the experiment.

The EEG and EMG signals were amplified 500 times and bandpass-filtered (0.1–1,000 Hz for EEG; 10–1,000 Hz for EMG; Model 3000, A-M Systems; DAM50, World Precision Instruments). The analysis was performed using in-house software developed in MATLAB R2020 (MathWorks). The EEG signals were subjected to Fast Fourier transform analysis from 1 to 60 Hz.

#### Detection of typical SWDs in PV-ArchT mice

SWDs were visually identified using EEG/EMG and video recordings. A previous study (Abdelaal et al., 2022) described the criteria for SWD events in PV-ArchT mice as repetitive spike-and-wave complexes associated with behavioral arrest lasting >0.5 s and ranging from 7 to 10 Hz. The average power of SWDs was double that of the EEG background.

#### Detection of other epileptiform discharges in PV-ArchT mice

**Figure 1.**
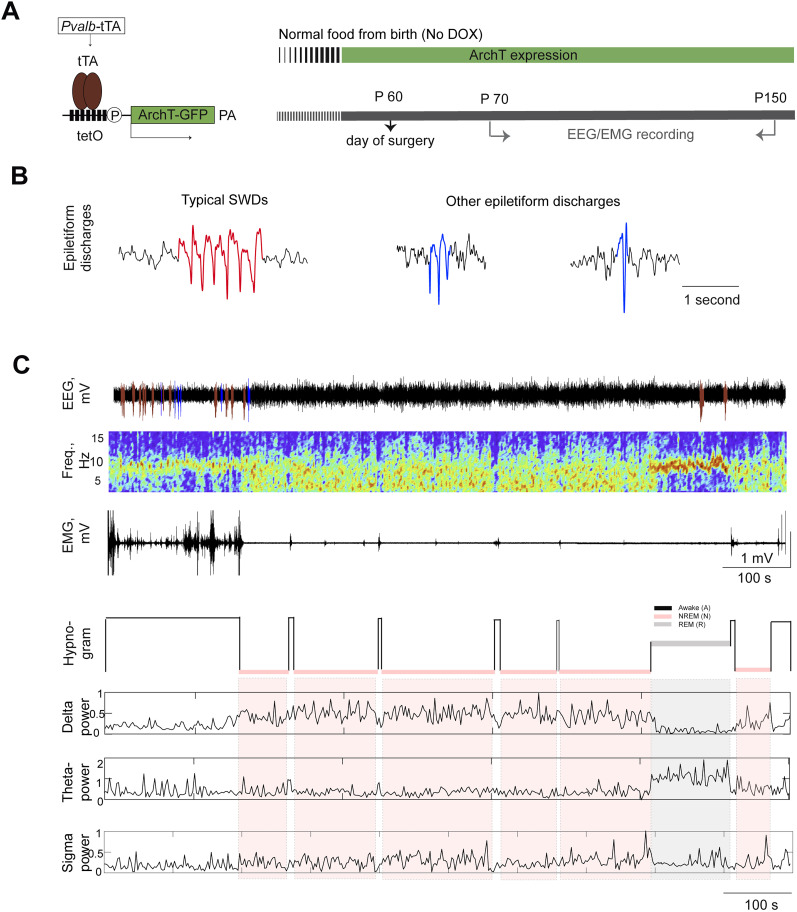
Detection of SWDs during awake–sleep stages in PV-ArchT mice. ***A***, Left, Schematic representation of PV-tTA::tetO-ArchT transgenic mice fed with normal food from birth; right, a timeline of ArchT expression and EEG/EMG recordings. ***B***, Types of epileptiform discharges in PV-ArchT mice. ***C***, Example of EEG recording on P120. From top to bottom, Cortical EEG (red and blue traces illustrate typical SWDs and other epileptiform discharges, respectively), EEG spectrogram, EMG, hypnogram, and powers of the delta, theta, and sigma bands.

Epileptiform discharges other than typical SWDs were visually identified from the EEG/EMG data based on specific criteria: spikes (sharp deflections) and spike–wave complexes that did not exhibit the typical features characteristic of SWDs (Abdelaal et al., 2022; Fig. 1*B*).

#### Detection of SWDs in GBL-treated mice

Since GBL-treated animals are known to display SWDs with lower frequencies compared with other animal models with robust behavioral arrest (Ishige et al., 1996), we used the following criteria for the SWD detection: repetitive spike-and-wave complexes associated with behavioral arrest lasting >0.5 s and ranging from 4 to 6 Hz (Ban et al., 1967; Venzi et al., 2015).

#### Detection of behavioral arrest during SWDs

Epileptic behavioral arrest was identified through simultaneous EEG/EMG and video monitoring. During the appearance of SWDs in the EEG, the mice abruptly and temporarily ceased their ongoing behavior, accompanied by very low EMG amplitudes. Upon termination of the SWDs, the mice's behavior resumed to normal, along with normal EMG amplitudes.

#### Detection of wake–sleep stages

EEG/EMG recordings with simultaneous video monitoring were conducted continuously for >24 h at a time. Sleep–wakefulness stages—wakefulness, NREM sleep, and REM sleep—were categorized from the EEG/EMG recordings through off-line analysis of 1 s epochs, following established methodologies described in previous studies (Ahnaou and Drinkenburg, 2011; Funato et al., 2016; Kato et al., 2022; Rayan et al., 2022). The EEG power spectra were averaged over the three frequency bands: delta (0.5–4.0 Hz), theta (6–9 Hz), and sigma (10–16 Hz). Wakefulness was scored based on low amplitude, fast EEG activity, and high-amplitude EMG signals. NREM sleep was identified based on high-power delta band and low-amplitude EMG signals. REM sleep was identified based on high-power theta band (6–9 Hz) signals and EMG atonia. The duration of each sleep–wake stage was aggregated every 3 h and utilized for the 24 h timeline assessment, encompassing both the 12 h light and dark phases.

#### Detection of sleep spindles

For sleep spindle analysis during NREM sleep, NREM sleep periods were identified in each mouse beforehand, as described previously. For each mouse, NREM sleep periods of 30 min were extracted from 2 to 4 h recordings, during which sleep spindles were detected and used for analysis. The sleep spindle detection method was based on previous studies (Fernandez and Lüthi, 2020; Osorio-Forero et al., 2021). That is, the raw data were filtered from the cortical EEG signal using a sigma band (10–16 Hz) filter, squared the resulting signal, and applied a threshold of 1.5 times the standard deviation above the mean values observed during NREM sleep. All peaks crossing this threshold were detected, and the endpoints of the events were extended to the nearest zero-crossing points before and after the threshold. Once sleep spindles were detected in NREM sleep periods, the following features were extracted: amplitude, which corresponds to the maximum value of the filtered signal within the spindle events; density, which is defined as the number of spindles divided by the unit of time; and duration, which is calculated as the time between the beginning and end of the spindle event (Table 1).

**Table 1.** Basic features of SWDs and sleep spindles

	SWDs (in PV-ArchT mouse)	Sleep spindle
Detection methods	Visually	Automated
Shape	SWDs	Waxing and waning
Power	7.3 ± 1.0 mV^2^ background, 3.5 ± 0.7 mV^2^	3.9 ± 0.3 mV^2^
Duration	0.5–10 s	0.5–1.5 s
Occurrence	During wakefulness, NREM sleep, and REM sleep	Only during NREM sleep
Frequency	7–10 Hz	10–16 Hz

SWDs, spike-and-wave discharges; PV-ArchT, parvalbumin-tetracycline transactivator; NREM, nonrapid eye movement; REM, rapid eye movement.

### Statistical analysis

All experiments were analyzed using MATLAB R2020 (MathWorks), Excel (Microsoft 365), and IBM SPSS Statistics 23. Mean values are reported for all numerical data, with error bars showing the standard error of the mean unless specified otherwise. Parametric tests, such as paired and two-tailed Student's *t* tests, one-way analysis of variance (ANOVA), and repeated-measure ANOVA followed by a Tukey–Kramer post hoc test, were used for the analysis. A *p* value of <0.05 was considered statistically significant (*p ≤ 0.05, **p ≤ 0.01, and ***p ≤ 0.001).

### Data availability

The corresponding author can provide the datasets upon reasonable request to support this study.

## Results

### PV-ArchT mice exhibited SWDs during both wakefulness and sleep stages

**Figure 2.**
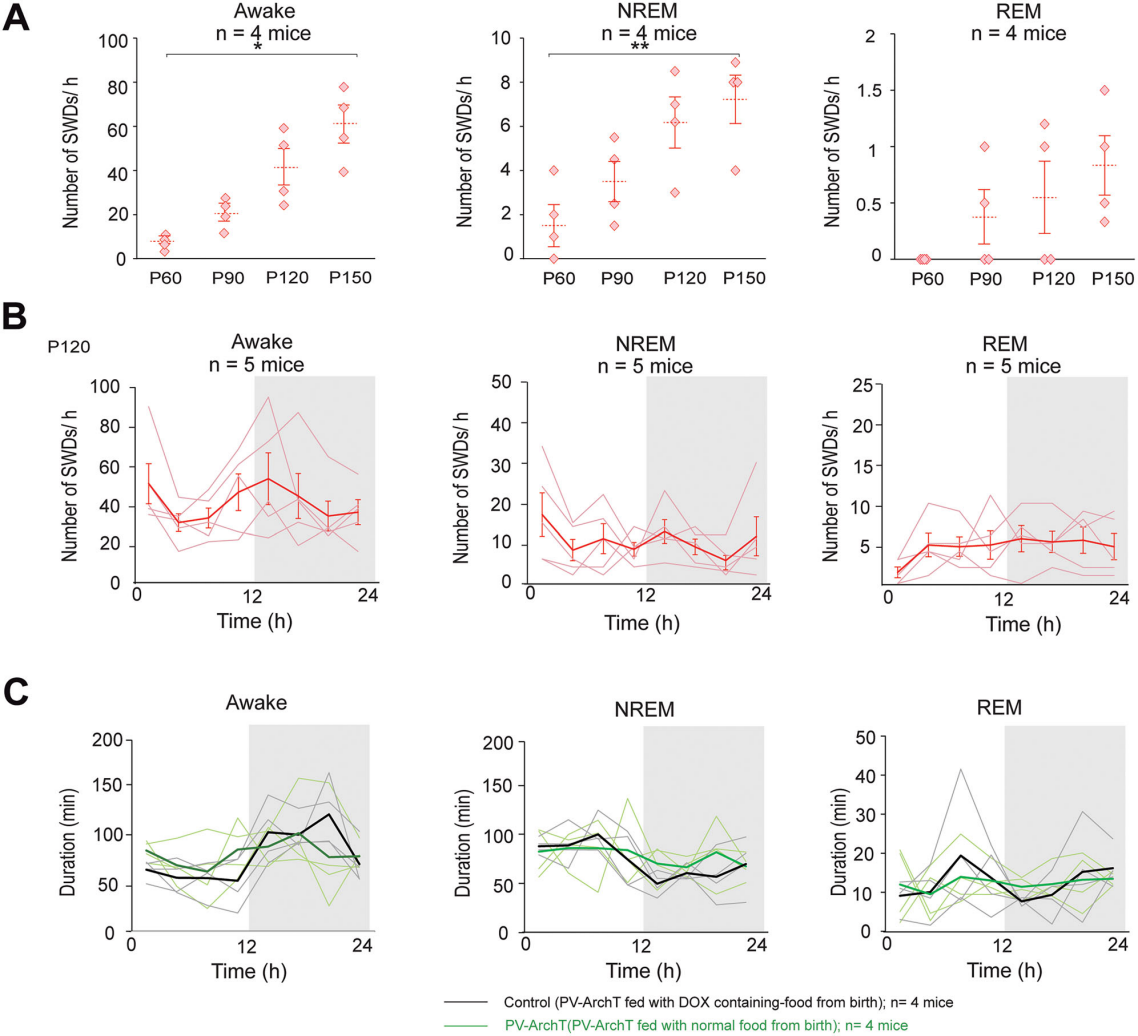
PV-ArchT mice exhibited SWDs during both the awake and sleep stages. ***A***, Dot plots show an increase in the number of SWDs with age during wakefulness, NREM sleep, and REM sleep (one-way ANOVA, awake, *n* = 4 mice; *F*_(3,12)_ = 3.82; *p* = 0.04; NREM sleep, *n* = 4 mice; *F*_(3,12)_ = 6.26; *p* = 0.008; REM sleep, *n* = 4 mice; *F*_(3,12)_ = 2.12; *p* = 0.15). Repeated-measure ANOVA followed by the Tukey–Kramer post hoc test, and the results of statistical tests are shown in Table 2. Data were recorded for 2–4 h for each mouse. ***B***, Line plots on P120 show no changes in the frequency of SWDs between the dark and light phases (one-way ANOVA; awake, *n* = 5 mice; *F*_(7,32)_ = 0.92; *p* = 0.50; NREM sleep, *n* = 5 mice; *F*_(7,32)_ = 1.05; *p* = 0.41; REM sleep, *n* = 5 mice; *F*_(7,32)_ = 0.83; *p* = 0.57). ***C***, Total duration of the awake, NREM, and REM sleep stages; data were recorded in 24 h with a 12 h light/dark cycle (two-way ANOVA; awake, *n* = 4 mice; *F*_(7,48)_ = 1.43; *p* = 0.22; NREM sleep, *n* = 4 mice; *F*_(7,48)_ = 0.69; *p* = 0.68; REM sleep, *n* = 4 mice; *F*_(7,48)_ = 0.37; *p* = 0.92). PV-ArchT mice fed with DOX-containing food from birth were used as controls.

The PV-ArchT mice were previously reported to display an absence seizure phenotype and fulfilled the criteria for face and pharmacological validities (Abdelaal et al., 2022). When the PV-ArchT mice were fed normal food (without DOX; Fig. 1*A*), the inhibitory opsin ArchT was highly expressed in the PV neurons of the TRN. Adult PV-ArchT mice fed normal food from birth exhibited cortical SWDs and other epileptiform discharges (Fig. 1*B*). The typical SWDs were characterized by 7–10 Hz repetitive activity lasting >0.5 s (Table 1). The power of SWDs exceeded that of the EEG background (*n* = 5 mice; 7.3 ± 1.0 vs 3.5 ± 0.7 mV^2^; *t*_(4) _= 6.2; *p* = 0.002). With age, both the duration and frequency of SWDs increased (Abdelaal et al., 2022). In this study, the distribution of SWD events during both awake and sleep stages was examined in PV-ArchT mice fed normal food (Fig. 1*C*). Vigilance stages were classified into wakefulness, NREM sleep, and REM sleep, and the number of SWDs in each stage was quantified according to the criteria described in the Materials and Methods section (Table 1). SWDs predominantly occurred during wakefulness and the transition from awake to NREM sleep stages. The occurrence of SWD events was 64.3% during the awake stage, 31.2% during the transition from awake to NREM sleep (categorized as NREM sleep), and 4.51% during REM sleep. The frequency of SWDs during wake, NREM sleep, and REM sleep remained consistent at 7–10 Hz. However, the duration of SWDs during REM sleep was shorter than that during wakefulness or NREM sleep (wake, 2.9 ± 0.7 s; NREM sleep, 3.0 ± 0.3 s; REM sleep, 1.9 ± 0.5 s). Furthermore, the number of SWDs increased significantly with age in the awake and NREM sleep stages (Fig. 2*A*, Table 2).

**Table 2.** Results of Tukey–Kramer post hoc test for the number of SWDs per hour with age during awake and NREM sleep for the data in Figure 2*A*

		Awake (*n* = 4 mice)	NREM (*n* = 4 mice)
P60	P90	0.50	0.50
	P120	0.01*	0.03*
	P150	<0.001***	0.01*
P90	P60	–	–
	P120	0.10	0.30
	P150	0.003**	0.10
P120	P60	–	–
	P90	–	–
	P150	0.18	0.1

The bold values indicate statistically significant *p* values.

*p ≤ 0.05, **p ≤ 0.01, ***p ≤ 0.001.

Next, whether PV-ArchT mice exhibited changes in the incidence of SWDs during the light/dark phases was investigated. EEG/EMG signals were recorded for 24 h, and the number of SWDs during the awake, NREM sleep, and REM sleep stages was quantified. No significant diurnal change was observed in the number of SWDs (Fig. 2*B*). Additionally, whether the SWD development in PV-ArchT mice caused changes in sleep duration was investigated, as it is well known that seizures disrupt sleep regulation (Sitnikova, 2021; Lehner et al., 2022), and 24 h EEG/EMG recordings were used to quantify the periods of wakefulness, NREM sleep, and REM sleep. The diurnal variations in wakefulness, NREM sleep, and REM sleep duration in PV-ArchT mice were not different from those in control mice (Fig. 2*C*). These results indicate that SWDs in PV-ArchT mice occurred in all sleep–wake stages regardless of light/dark phases.

### SWD development is associated with sleep spindle alterations in PV-ArchT mice

**Figure 3.**
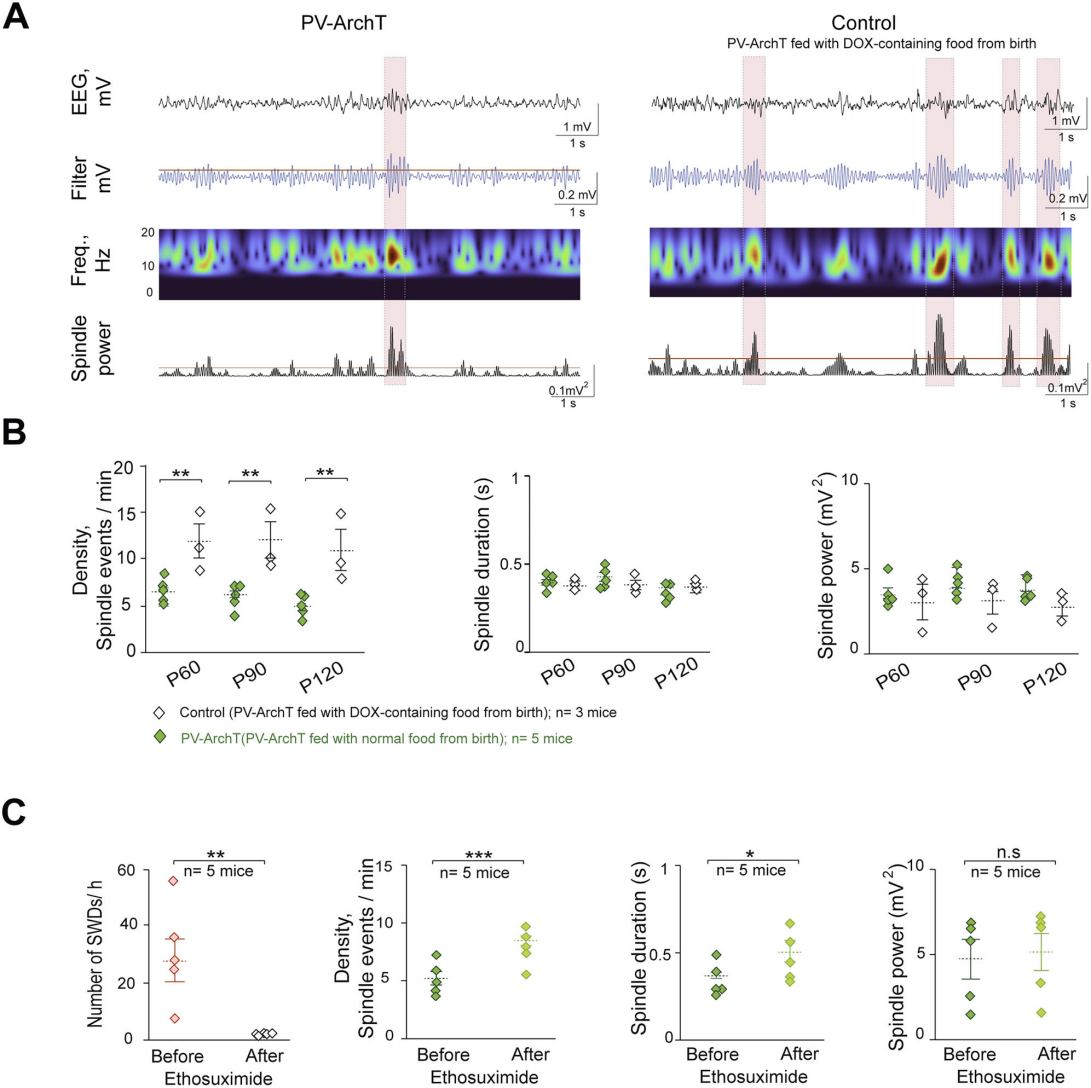
Relationship between sleep spindle alteration and SWD development in PV-ArchT mice. ***A***, Method for detecting sleep spindles in PV-ArchT (left) and control (right, PV-ArchT mice fed with DOX-containing food from birth) mice. From top to bottom, Raw EEG data recording, filtered sigma signals, wavelet analysis, and spindle power. The red lines represent the threshold value. Red-shaded areas indicate sleep spindle events. ***B***, Summary graphs from left to right showing the sleep spindle patterns (density, duration, and power, respectively), comparing PV-ArchT mice (*n* = 5 mice for each group) with control mice (*n* = 3 mice for each group; PV-ArchT mice fed with DOX-containing food all life were used as controls). The results of statistical tests are shown in Table 3. ***C***, Summary graphs from left to right show the number of SWDs/h and the sleep spindle patterns (density, duration, and power) before and after ethosuximide injection (*n* = 5 mice).

The CTC network, including TRN neurons, has been implicated in the development of SWDs, as well as in physiological oscillations like sleep spindles (Steriade et al., 1993; Snead, 1995; McCormick and Bal, 1997; Pinault and O’Brien, 2005). This study aimed to investigate whether SWDs are linked to sleep spindle dysfunction, given their established connection. Specifically, the alteration of sleep spindle patterns was investigated in PV-ArchT mice during the development of SWDs compared with that in control mice. Our control group consisted of PV-ArchT mice fed with DOX-containing food from birth (Fig. 3*A*). Our results revealed a reduction in sleep spindle density in PV-ArchT mice compared with the control group. Specifically, when examining changes in sleep spindle density between Postnatal Day (P)60 and P120, a decline was observed (Fig. 3*B*, Table 3). However, the duration and power of the sleep spindle did not differ from those of the control group and remained unchanged with age (Fig. 3*B*, Table 3).

**Table 3.** Results of the average sleep spindle patterns (density, duration, and power, respectively), with age in PV-ArchT and control mice for the data in Figure 3*B*

	Density	Duration	Power
P60	6.44 ± 0.55 vs 11.9 ± 1.84	0.39 ± 0.02 vs 0.37 ± 0.002	3.39 ± 0.42 vs 3 ± 1.03
	*p* = 0.01**	*p* = 0.33	*p* = 0.68
P90	6.20 ± 0.27 vs 12.0 ± 1.97	0.43 ± 0.03 vs 0.38 ± 0.03	3.77 ± 0.38 vs 3.13 ± 0.82
	*p* = 0.01**	*p* = 0.24	*p* = 0.44
P120	4.94 ± 0.33 vs 10.9 ± 2.26	0.36 ± 0.009 vs 0.35 ± 0.007	3.72 ± 0.22 vs 2.76 ± 0.55
	*p* = 0.01**	*p* = 0.83	*p* = 0.13

Control mice are PV-ArchT mice fed with DOX-containing food from birth; *n* = 3 mice. PV-ArchT mice fed with normal food from birth; *n* = 5 mice.

The bold values indicate statistically significant p values.

**p ≤ 0.01.

The effect of antiepileptic treatment on sleep spindles using ethosuximide (Glauser et al., 2013; Brigo and Igwe, 2017; Kozák et al., 2020), which is the first-line treatment for absence seizures in humans in addition to the established genetic rodent models WAG/Rij and GAERS, was also investigated. Ethosuximide was administered to epileptic PV-ArchT mice at a dose of 100 mg/kg intraperitoneally. After 30 min of a single dose of ethosuximide, the number of SWD events was markedly reduced (30–180 min after injection; *n* = 5 mice; 28.5 ± 7.47 vs 1.76 ± 0.66 SWDs/h; *t*_(4) _= 3.35; *p* = 0.01; Fig. 3*C*). Additionally, both density and duration of sleep spindles were increased after ethosuximide injection (30–180 min after injection; *n* = 5 mice; density, 5.13 ± 0.61 vs 8.25 ± 0.38 spindle events/min; *t*_(4) _= −8.43.1; *p* = 0.0005; duration, 0.37 ± 0.02 vs 0.50 ± 0.07 s; *t*_(4) _= −2.30; *p* = 0.04; Fig. 3*C*). There was no significant change observed in sleep spindle power (30–180 min after injection; *n* = 5 mice; power, 4.96 ± 1.13 vs 5.42 ± 1.11 mV^2^; *t*_(4) _= −1.47; *p* = 0.11; Fig. 3*C*). These results suggest a potential link between the dynamics of SWDs and alterations in sleep spindles due to the manipulation of TRN-PV neurons and therapeutic interventions.

### Chronological order of reduction in sleep spindle density and SWD onset

**Figure 4.**
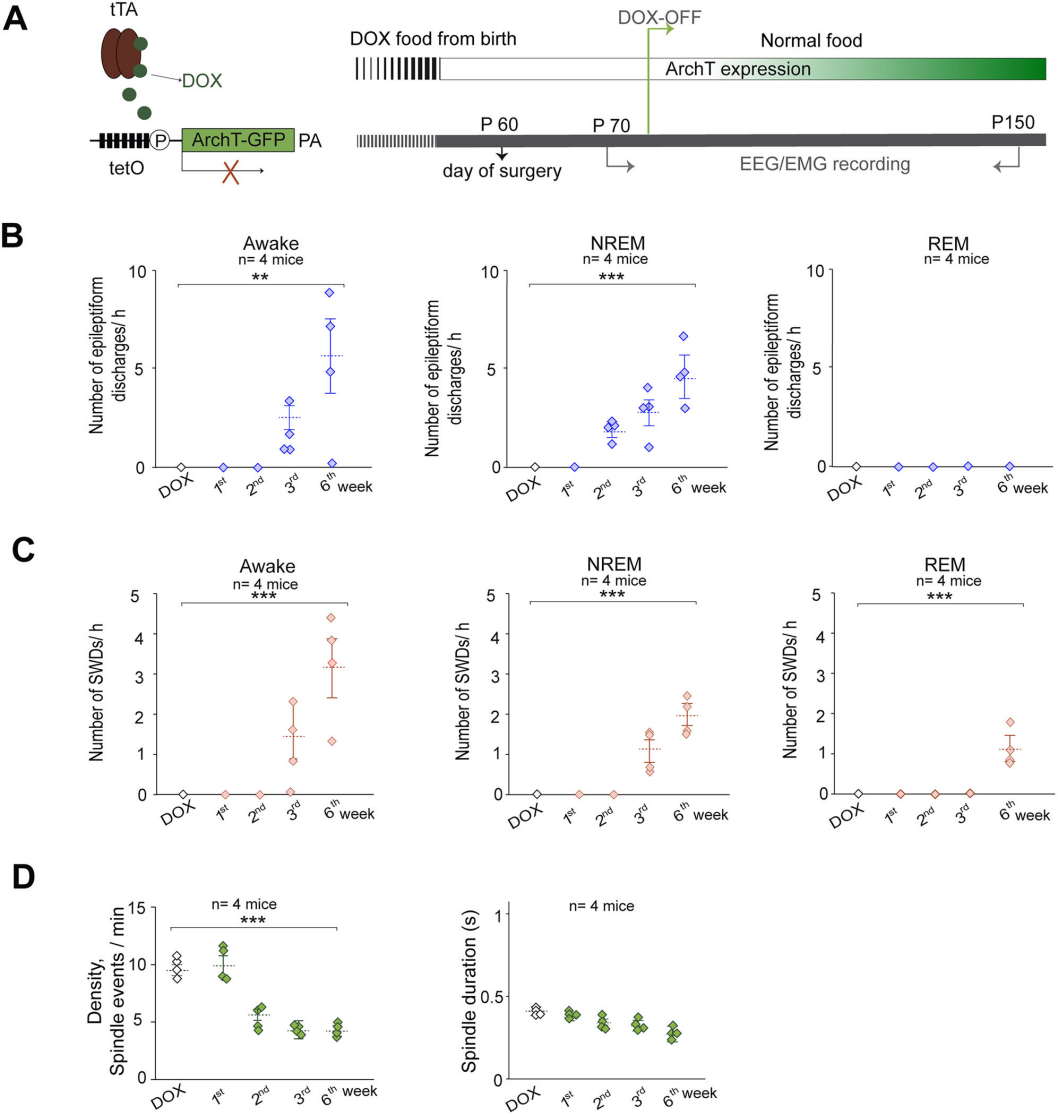
Temporal sequence between the SWD development and sleep spindle patterns in PV-ArchT mice. ***A***, Left, Schematic representation of PV-tTA::tetO-ArchT transgenic mice fed with DOX-containing food. Right, A timeline of ArchT expression and EEG/EMG recordings. ***B***, Dot plots showing the development of other epileptiform discharges during the awake, NREM, and REM stages (one-way ANOVA; awake, *n* = 4 mice; *F*_(4,15)_ = 5.96; *p* = 0.004; NREM sleep, *n* = 4 mice; *F*_(4,15)_ = 13.7; *p* < 0.001). Repeated-measure ANOVA followed by the Tukey–Kramer post hoc test, and the results of statistical tests are shown in Table 4. ***C***, Dot plots showing the development of typical SWDs during the awake, NREM, and REM stages (one-way ANOVA; awake, *n* = 4 mice; *F*_(4,15)_ = 10.2; *p* = 0.0003; NREM sleep, *n* = 4 mice; *F*_(4,15)_ = 34.2; *p* < 0.001; REM sleep, *n* = 4 mice; *F*_(4,15)_ = 37.3; *p* < 0.001). Repeated-measure ANOVA followed by the Tukey–Kramer post hoc test, and the results of statistical tests are shown in Table 5. ***D***, Dot plots showing changes in sleep spindle density and duration (one-way ANOVA; density, *n* = 4 mice; *F*_(4,15)_ = 49.8; *p* < 0.001; duration, *n* = 3 mice; *F*_(4,15)_ = 1.53; *p* = 0.24). Repeated-measure ANOVA followed by the Tukey–Kramer post hoc test, and the results of statistical tests are shown in Table 6.

As the presence of SWDs was associated with a decrease in sleep spindle density, we aimed to determine whether the decrease in sleep spindle density precedes or follows the emergence of SWDs. To assess temporal changes, EEG/EMG monitoring of PV-ArchT mice before and after the withdrawal of DOX-containing food (DOX-off) was conducted. This manipulation induced the expression of ArchT in PV-TRN neurons, resulting in the induction of SWDs (Abdelaal et al., 2022). By initially providing DOX-containing food to PV-ArchT mice from birth and subsequently switching to normal food (Fig. 4*A*), the SWD's development with EEG/EMG/video monitoring over time was evaluated. The occurrence of typical SWDs, epileptiform discharges other than SWDs, and sleep spindle alterations before and after DOX discontinuation was examined. The same parameters were used to quantify the SWDs, as previously described in the Materials and Methods section. Initially, during Week 2 after DOX discontinuation, epileptiform discharges instead of the typical SWDs that occurred during the NREM sleep was observed. By Week 3, these epileptiform discharges had spread to the awake stage (Fig. 4*B*). It was only during Week 3 that typical SWDs were detected (Fig. 4*C*). Over time, the number of epileptiform discharges and typical SWD events increased with high ArchT expression (Fig. 4*B*,*C*; Tables 4, 5, respectively). Notably, sleep spindle density began to decrease in Week 2 after DOX discontinuation, which coincided with the occurrence of epileptiform discharges and preceded the onset of typical SWDs in Week 3 (Fig. 4*D*). However, from Weeks 2 to 6, sleep spindle density remained consistently reduced without further decrease (Fig. 4*D*, Table 6). Additionally, no significant changes in the duration of sleep spindles before and after discontinuing DOX were noted (Fig. 4*D*). These findings suggest a specific temporal relationship in which a reduction in sleep spindle density precedes the onset of typical SWDs.

**Table 4.** Results of Tukey–Kramer post hoc test the number of epileptiform discharges per hour during awake and NREM sleep for the data in Figure 4*B*

		Awake (*n* = 4 mice)	NREM (*n* = 4 mice)
DOX	Week 1	1.0	0.9
	Week 2	1.0	0.2
	Week 3	0.5	0.01**
	Week 6	0.01**	<0.001***
Week 1	DOX	-	-
	Week 2	1.00	0.2
	Week 3	0.5	0.02*
	Week 6	0.01**	<0.001***
Week 2	DOX	-	-
	Week 1	-	-
	Week 3	0.5	0.6
	Week 6		0.01**
Week 3	DOX	-	-
	Week 1	-	-
	Week 2	-	-
	Week 6	0.1	0.1

The bold values indicate statistically significant *p* values.

*p ≤ 0.05, **p ≤ 0.01, ***p ≤ 0.001.

**Table 5.** Results of the Tukey–Kramer post hoc test on the number of SWDs per hour during awake and NREM sleep for the data are in Figure 4*C*

		Awake (*n* = 4 mice)	NREM (*n* = 4 mice)	REM (*n* = 4 mice)
DOX	Week 1	1.0	1.0	1.0
	Week 2	1.0	0.004	1.0
	Week 3	0.2	<0.001***	**1.0**
	Week 6	0.001***	<0.001***	<0.001***
Week 1	DOX	-	-	-
	Week 2	1.0	0.004	1.0
	Week 3	0.169	<0.001***	**1.0**
	Week 6	0.001***	<0.001***	<0.001***
Week 2	DOX	-	-	-
	Week 1	-	-	-
	Week 3	0.169	0.027	1.0
	Week 6	0.001***	<0.001***	<0.001***
Week 3	DOX	-	-	-
	Week 1	-	-	-
	Week 2	-	-	-
	Week 6	0.10	0.18	<0.001***

The bold values indicate statistically significant *p* values.

***p ≤ 0.001.

**Table 6.** Results of Tukey–Kramer post hoc test for the changes in sleep spindle density for the data in Figure 4*D*

		Density (*n* = 4 mice)
DOX	Week 1	0.87
	Week 2	<0.001***
	Week 3	<0.001***
	Week 6	<0.001***
Week 1	DOX	–
	Week 2	<0.001***
	Week 3	<0.001***
	Week 6	<0.001***
Week 2	DOX	–
	Week 1	–
	Week 3	0.15
	Week 6	0.10
Week 3	DOX	–
	Week 1	–
	Week 2	–
	Week 6	0.99

The bold values indicate statistically significant *p* values.

***p ≤ 0.001.

### Temporal relationship between sleep spindles and SWDs in pharmacological absence seizure models

**Figure 5.**
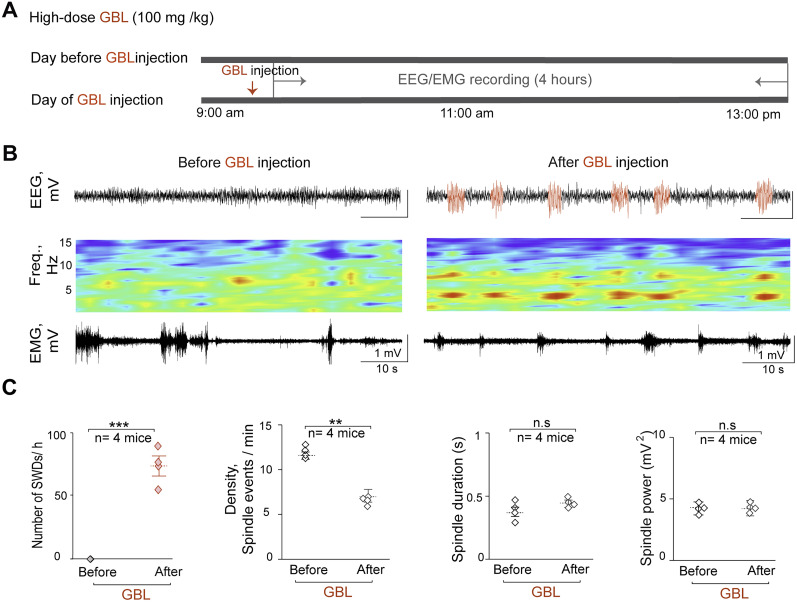
Drug-induced SWDs are associated with a reduction in sleep spindle density. ***A***, Timeline illustrating the administration of GBL (100 mg/kg) to wild-type mice. ***B***, Example of an EEG recording before (left) and after (right) GBL injection. From top to bottom, Cortical EEG, EEG spectrogram, and EMG. Red traces indicate SWDs. ***C***, Summary graphs from left to right show the number of SWDs/h and the sleep spindle patterns (density, duration, and power) before and after GBL administration (*n* = 4 mice).

Next, whether the temporal relationship between SWD development and sleep spindle alterations observed in our genetic model of absence seizures (PV-ArchT) could also be observed in other pharmacological models was investigated. GBL was first administered to wild-type mice at a dose of 100 mg/kg during the light phase (Hodor et al., 2015; Venzi et al., 2015; Fig. 5*A*), which successfully induced SWDs with a frequency of 4–6 Hz (*n* = 4 mice; SWD events, 73.4 ± 6.82 SWDs/h; *p* = 0.001; Fig. 5*B*). The SWDs began to appear 4.2 ± 0.5 min after the injection of GBL. The average duration of these SWDs was 3.3 ± 0.1 s. Although the GBL has sedative/hypnotic effects, a dose below 200 mg/kg does not significantly increase sleep time during the light phase; it only increases the power of slow waves (Van Sassenbroeck et al., 2001; Felmlee et al., 2010; Spano et al., 2022). The changes in sleep spindle patterns during NREM sleep before and after the GBL injection were examined. A significant reduction was noted in sleep spindle density during the first 4 h after the GBL administration (*n* = 4 mice; density, 11.6 ± 0.59 vs 6.98 ± 0.73 spindle events/min; *t*_(3) _= 3.82; *p* = 0.01; Fig. 5*C*). No changes were observed in the power and duration of sleep spindles before and after the GBL injection (*n* = 4 mice; duration, 0.37 ± 0.04 vs 0.43 ± 0.03 s; *t*_(3) _= −1.89; *p* = 0.08; power, 4.26 ± 0.05 vs 4.15 ± 0.13 mV^2^; *t*_(3) _= 0.76; *p* = 0.25; Fig. 5*C*). These results are consistent with the findings that in PV-ArchT mice, the SWD development was associated with a decrease in sleep spindle density.

**Figure 6.**
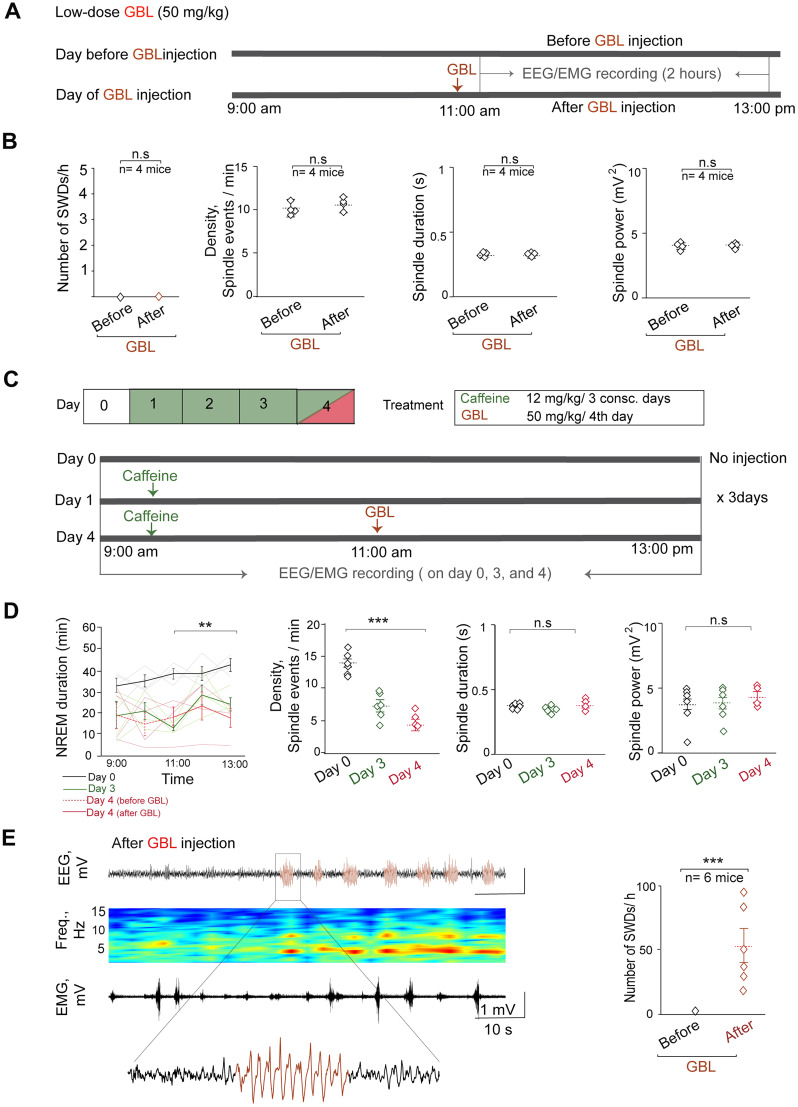
Temporal relationship between sleep spindle and SWDs in the GBL model of absence seizure. ***A***, Timeline illustrating the administration of GBL (50 mg/kg) to wild-type mice. ***B***, Summary graphs from left to right show the number of SWDs/h and the sleep spindle patterns (density, duration, and power) before and after GBL administration (*n* = 4 mice). ***C***, The panels illustrate the daily protocols for caffeine and GBL injections. ***D***, Summary graphs from left to right show the time course of NREM sleep duration per hour (one-way ANOVA from 11:00 to 13:00, *F*_(2,9)_ = 12.2; *p* = 0.002) and sleep spindle patterns (density, duration, and power) on Days 0, 3, and 4 (one-way ANOVA; density, *F*_(2,13)_ = 20.3; *p* = 0.00009; duration, *F*_(2,13)_ = 4.45; *p* = 0.33; power, *F*_(2,13)_ = 0.12; *p* = 0.89). Note that, for Days 0 and 3, data from NREM sleep between 9:00 and 13:00 were used for analysis, whereas for Day 4, data from NREM sleep between 11:00 and 13:00 were used for analysis. Repeated-measure ANOVA followed by the Tukey–Kramer post hoc test, and the results of statistical tests are shown in Tables 7 and 8. ***E***, Left, Example of an EEG recording after GBL injection. From top to bottom, Cortical EEG, EEG spectrogram, and EMG. Red traces indicate SWDs. Right, Dot plot showing the number of SWDs before and after GBL injection (*n* = 6 mice).

Next, the temporal relationship in which sleep spindle alterations occur before the onset of SWDs was determined. Initially, a low dose of GBL (50 mg/kg) was administered, which proved insufficient to induce SWD or to increase sleep time in wild-type mice (Ishige et al., 1996; Venzi et al., 2015). Following the injection of 50 mg/kg GBL into wild-type mice (Fig. 6*A*), no SWDs were observed in these mice. Additionally, there were no significant changes observed in sleep spindle density, duration, and power during the first 2 h after administration of low dose of GBL (density, 10.2 ± 0.60 vs 10.6 ± 0.52 spindle events/min; *t*_(3) _= −0.51; *p* = 0.66; duration, 0.31 ± 0.01 vs 0.32 ± 0.01 s; *t*_(3) _= −0.51; *p* = 0.66; power, 3.85 ± 0.21 vs 3.94 ± 0.21 mV^2^; *t*_(3) _= −2.36; *p* = 0.14; Fig. 6*B*).

Then, caffeine at a dose of 12 mg/kg was repeatedly administered to wild-type mice to induce a reduction in sleep spindles (Fig. 6*C*). This dose is known to promote wakefulness and decrease sleep duration in mice (Lazarus et al., 2011). On Day 1, the mice were unable to sleep after the caffeine injection at 9:00 A.M. in the light phase. However, after injecting caffeine for 3 consecutive days, the mice were able to sleep even after caffeine administration.

The duration of NREM sleep per hour from 9:00 A.M. to 13:00 was evaluated. On Day 3 of caffeine injection, the NREM sleep duration per hour decreased compared with that at the same time of day before the caffeine administration (Day 0; 11:00 A.M.–13:00; Day 0 vs Day 3, *n* = 4 mice; NREM sleep duration; 39.2 ± 0.84 vs 21.5 ± 1.92 min; Fig. 6*D*, Table 7). Additionally, a decrease in sleep spindle density after the caffeine injection was noted (Day 0 vs Day 3, 13.7 ± 1.33 vs 7.13 ± 0.79 spindle events/min; Fig. 6*D*, Table 8). However, the duration and power of the sleep spindles did not change (Day 0 vs Day 3, *n* = 6 mice; duration, 0.37 ± 0.007 vs 0.34 ± 0.008 s; power, 4.02 ± 0.62 vs 3.84 ± 0.57 mV^2^; Fig. 6*D*). On Day 4, 50 mg/kg GBL was administered to the mice for 2 h after repeated caffeine administration (Fig. 6*C*). The additional GBL administration did not significantly alter the duration of NREM sleep (11:00 A.M.–13:00; Day 4 vs Day 3, *n* = 4 mice; NREM sleep duration; 21.5 ± 1.92 vs 19.3 ± 4.97 min; Fig. 6*D*, Table 7). Notably, typical SWDs were observed in mice administered low-dose GBL after repeated caffeine administration (*n* = 6 mice; SWD number, 53 ± 13.2; Fig. 6*E*). The sleep spindle density, which was reduced by repeated caffeine administration for 3 d, remained unchanged with the additional administration of low-dose GBL (from 11:00 A.M. to 13:00; *n* = 4 mice; density, 4.46 ± 0.82 spindle events/min; duration, 0.39 ± 0.021 s; power, 4.27 ± 0.44 mV^2^; Fig. 6*D*, Table 8). These results confirm the temporal relationship between sleep spindle alterations and SWD development under pharmacological induction. Additionally, they suggest that the decline of sleep spindle density may facilitate the development of SWDs.

**Table 7.** Results of Tukey–Kramer post hoc test for the changes in time course of NREM sleep duration per hour from 11:00 to 13:00 for the data in Figure 6*D*

		NREM duration (*n* = 4 mice)
Before caffeine (Day 0)	After caffeine (Day 3)	<0.008**
	Caffeine + GBL (Day 4)	<0.004**
After caffeine (Day 3)	Before caffeine (Day 0)	–
	Caffeine + GBL (Day 4)	0.88

The bold values indicate statistically significant *p* values.

**p ≤ 0.01.

**Table 8.** Results of Tukey–Kramer post hoc test for the changes in sleep spindle density on Days 0, 3, and 4 for the data in Figure 6*D*

		Density
Before caffeine (Day 0)	After caffeine (Day 3)	<0.001***
	Caffeine + GBL (Day 4)	<0.001***
After caffeine (Day 3)	Before caffeine (Day 0)	-
	Caffeine + GBL (Day 4)	0.32

Before/after caffeine (*n* = 6 mice), caffeine + GBL (*n* = 4 mice).

The bold values indicate statistically significant *p* values.

***p ≤ 0.001.

## Discussion

This study provides insights into the temporal relationship between sleep spindle alterations and the occurrence of SWDs in both genetic and pharmacological animal models of absence seizures: a reduction in sleep spindle density preceded the occurrence of SWDs. This suggests that the changes in the sleep spindle may have potential predictive value for the occurrence of SWDs. Furthermore, these changes may also play a role in the development of SWDs.

### Distribution of SWDs across wake–sleep stages

The occurrence of SWDs demonstrates a clear link with alertness levels, occurring more frequently during wakefulness and NREM sleep in absence seizure patients (Posner, 2013). SWDs are commonly observed during the N2 stage of NREM sleep. These events are characterized by a 3 Hz frequency and a duration of >2 s, similar to patterns observed during wakefulness, but often display greater disorganization (Chen et al., 2022). The occurrence of SWDs during sleep, especially in children, may indicate drug resistance or pose diagnostic challenges (Sadleir et al., 2011). Notably, SWDs are rarely detected during REM sleep. However, clinical studies on SWDs during sleep are limited, prompting researchers to rely on animal models. The distribution of SWDs in relation to the vigilance stage has been studied in genetic rat models, such as WAG/Rij rats and GAERS, feline models, and other mouse models that present spontaneous or paroxysmal SWDs. In these animals, SWDs were most prevalent during wakefulness and NREM sleep, with minimal occurrences during REM sleep (Lannes et al., 1988; Marescaux et al., 1992; Sitnikova et al., 2014; Funato et al., 2016; Sitnikova, 2021). Our PV-ArchT mice shared similarities with the previous models. In the PV-ArchT mice, SWDs were observed across all vigilance stages but were more frequent during wakefulness and transition from awake to NREM sleep. SWDs were rarely observed during REM sleep. SWDs have a different appearance from theta waves during phasic REM, characterized by increased frequency and amplitude, in that SWDs have spikes. Although they are easily distinguished visually, it might be difficult to completely exclude phasic REM from SWDs during REM sleep. Phasic REM sleep is typically identified by measuring REMs (Simor et al., 2020). However, simultaneous recording of eye movements in mice is technically challenging during continuous 24 h freely moving monitoring. Future studies could benefit from advanced recording techniques or algorithms to perfectly differentiate between these states. In summary, SWDs in both human and animal models exhibit vigilance-dependent characteristics and are influenced by the wake–sleep cycle.

### Distinct TRN activity involved in sleep spindle and SWD generation

The TRN, within the CTC network, regulates sleep spindles and SWDs through two distinct firing patterns: tonic and burst firing (Steriade et al., 1993; McCormick and Bal, 1997; Schönauer and Pöhlchen, 2018). These firing activities in the TRN are triggered by T-type Ca^2+^ channels, specifically the Cav3.2 and Cav3.3 isoforms. Notably, Cav3.3 and its associated burst firing, which are essential for sleep spindle initiation, may not be critical for SWD development (Crunelli and Leresche, 2002; Halassa et al., 2011). In Cav3.2 and Cav3.3 double-knock-out mice, the complete loss of burst firing and increase in tonic firing suppressed sleep spindles and induced SWD following systemic GBL injection (J. Lee et al., 2013; S. E. Lee et al., 2014; Lory et al., 2020). Based on these findings, Lee's group proposed that high tonic firing in the TRN may contribute to SWD formation (S. E. Lee et al., 2014). Furthermore, integration of our previous research findings with the current findings in PV-ArchT mice, which demonstrate SWD generation alongside a decline in sleep spindles and impaired TRN burst firing (Figs. 1–3; Abdelaal et al., 2022), supports the hypothesis that burst firing may not be essential for SWD generation. However, investigations into changes in tonic firing in the PV-ArchT model are still pending. Together, these observations suggest that different firing patterns in the TRN may drive alterations in sleep spindle activity and SWD generation. The precise mechanism by which the tonic firing of TRN controls the formation of sleep spindles and SWDs remains unclear. Addressing these questions holds the potential to open new avenues for innovative therapeutic approaches aimed at managing seizures and various sleep-related conditions.

### Reduction in sleep spindle density during absence seizures

Zhang et al. (2023) reported that children with absence seizures exhibited lower spindle density and duration during the N2 stage of NREM sleep than the control group. Furthermore, children with absence seizures and cognitive deficits display marked reductions in sleep spindles (Zhang et al., 2023). One notable challenge in their study of sleep spindles in patients with absence seizures was the potential confounding effect of antiseizure medication. Experimental models of absence seizures have been used to examine this relationship. However, information on changes in sleep spindles in monogenic mutant models of absence seizures, such as stargazer, tottering, and lethargic, is lacking. The only available data on this relationship come from the WAG/Rij rat model and Long–Evans rats, which showed that the development of SWDs is associated with changes in sleep spindle patterns (Leresche et al., 2012; Sitnikova et al., 2014, 2023; Kozák et al., 2020). These previous data support the idea that the development of SWDs is associated with a reduction in sleep spindle density. Building on the findings from PV-ArchT mice, this study contributes a novel perspective by uncovering the temporal relationship between SWD development and changes in sleep spindles. A decrease in sleep spindle density was noted 1 week before the onset of SWDs. These findings suggest that identifying absence seizures involves not only recognizing pathological oscillations such as spikes followed by waves but also noting the absence of physiological oscillations. Moreover, in the PV-ArchT mouse model, a chronological sequence of typical SWDs and other epileptiform discharges was observed. Our findings indicate that these other epileptiform discharges appear 1 week before the onset of typical SWDs, providing two significant insights. First, the occurrence of these epileptiform discharges before the SWD development could aid in early diagnosis and treatment. Second, investigating whether these epileptiform discharges and SWDs correlate with the level of ArchT expression in PV-TRN could elucidate whether they stem from abnormalities in the CTC network, including TRN neurons. This approach could deepen our understanding of the pathophysiology of absence seizures and illuminate the underlying mechanisms of these events.

### The role of sleep spindle in the SWD dynamics (pharmacological insights)

Acute pharmacological experiments have been performed to explore the reciprocal connection between sleep spindles and SWDs in WAG/Rij rats (Van Luijtelaar, 1997; Sitnikova and van Luijtelaar, 2005). For instance, barbiturates and benzodiazepines have been shown to enhance sleep spindles and concurrently suppress SWDs. Conversely, clonidine has been shown to aggravate SWDs in a dose-dependent manner and has been associated with a reduction in sleep spindles (Hirshkowitz et al., 1982; Van Luijtelaar, 1997; Sitnikova and van Luijtelaar, 2005). These findings provide evidence suggesting a shared involvement of CTC circuits in both sleep spindles and SWDs. The association between decreased sleep spindle density and the occurrence of SWDs was investigated. Notably, SWDs were induced in mice with lower-than-normal sleep spindle density using low-dose GBL. Previous studies have confirmed that the induction of SWDs by GBL is dose-dependent, with doses below 70 mg/kg being insufficient to induce SWDs in mice. Our observations indicate that mice with reduced sleep spindle density due to caffeine administration exhibited SWD upon being injected with a non-SWD–inducing dose of GBL (Ishige et al., 1996; Venzi et al., 2015). These findings underscore the importance of sleep spindles in SWD dynamics. However, there are limitations to our approach of using pharmacologically induced SWDs and alterations in the sleep spindles. First, we did not use a control group with saline injections. Even saline injections can affect sleep architecture and spindle activity due to the restraint stress with injection. However, we have not addressed this possibility in the present study. Second, caffeine exhibits dual effects on sleep spindles and overall sleep duration, complicating the interpretation of our results. While our study highlights a temporal relationship between reduced sleep spindle density and SWD onset, whether this reduction is the primary driver of SWDs or if changes in sleep duration also play a significant role cannot be definitively concluded. Third, the artificial induction of SWDs using GBL may not fully replicate the complexity of naturally occurring SWDs. Drug–drug interactions, such as those between caffeine and GBL, could also introduce confounding factors, potentially influencing SWD generation. Finally, our study primarily focused on short-term effects, and the long-term consequences or reversibility of changes in sleep spindle density following GBL administration were not explored. Future research should incorporate more naturalistic models and delve into the underlying neurobiological mechanisms. Direct manipulation of sleep spindles, such as through optogenetic techniques to trigger or suppress sleep spindles, could provide conclusive evidence on the prevention or induction of SWDs. Such investigations will be crucial for comprehensively understanding the bidirectional relationship between SWD development and sleep spindle density.

Enhanced understanding of the role of sleep spindle in SWD developments in the CTC circuit will help improve the treatment of absence seizure. Our finding of the crucial temporal link between sleep spindle reduction and the onset of SWDs paves the way for new approaches to early detection and intervention in absence seizures.

## Synthesis

Reviewing Editor: Jean Christophe Poncer, INSERM Decisions are customarily a result of the Reviewing Editor and the peer reviewers coming together and discussing their recommendations until a consensus is reached. When revisions are invited, a fact-based synthesis statement explaining their decision and outlining what is needed to prepare a revision will be listed below. The following reviewer(s) agreed to reveal their identity: Adrien Peyrache.

Both reviewers and I believe that this study presents compelling data supporting the idea that spindle density could serve as a predictor of epileptic SWD. While this information may be of interest to a specialized audience and potentially worthy of publication in eNeuro, the manuscript has several issues that need to be addressed before further editorial consideration. Below is a summary of the major points that require attention in a revised version of the manuscript.

1. A general comment from both reviewers is that the authors should tone down several of their conclusions to better reflect their actual observations. Specifically, the change in spindle density and the development of SWDs in several experimental conditions are intriguing but only correlative, not causal, as the authors suggest. The authors should carefully review and correct any overstatements throughout their manuscript.

2. Both reviewers have suggested that the tests used in the data from figures 2 and 3 are inappropriate and have recommended using repeated measure ANOVA instead. Reviewer 2 has also pointed out that some key conclusions of the paper rely on an insufficient number of observations. We strongly recommend that the authors strengthen their data and conclusions with at least a couple of additional observations and/or perform power analysis of their statistics. One reviewer identified inconsistencies between the number of data points in figures and the number of observations reported in the text. This issue must be fixed.

3. The primary effect of caffeine on sleep pressure and spindle density is ambiguous, and further discussion and additional control experiments are necessary to address this issue, as suggested by reviewer 2.

4. The authors' experimental protocols and analysis lack sufficient detail, particularly in regards to behavioral data.

6. It is unclear why the criteria for SWD detection differ between experimental paradigms. Please provide clarification.

--

Reviewer 1

The present study offers a thorough and innovative investigation into the temporal relationships between sleep spindle density and spike-and-wave discharges (SWDs) in a genetic mouse model of epilepsy as well as wild-type animals with pharmacological manipulation. Using the Pvalb-tetracycline transactivator (tTA)::tetO-ArchT double transgenic mouse and controlled onset experiments, this study showed that the decrease in spindle density occurred two weeks after the discontinuation of DOX- containing food, which starts ArchT expression, while SWD density increased a week later. Furthermore, the study shows that SWDs can be induced by a low dose of gamma-butyrolactone (GBL) only when administered after three days of caffeine injections that result in reduced spindle density. These findings confirm the now well accepted hypothesis that spindles and SWRs share the same underlying networks, and paves the way for potential predictive role of sleep spindle density alterations in SWD development. However, some of the conclusions regarding causality and direct relationship between decrease in spindles and emergence of SWDs hould be toned down.

Major comments

The most critical remark regarding this manuscript is that some of the conclusions should be toned down. Specifically, while the study compellingly demonstrates a temporal association and potential predictive value of sleep spindle density changes for SWD development, the claim of a direct causal relationship between decreased sleep spindle density and the activation of SWDs might be premature without direct manipulation of sleep spindles, such as through optogenetic triggering/suppressing, that conclusively shows the prevention or induction of SWDs. Reduction of sleep spindles was not the only consequences of caffeine injection. Instead, the animals were sleep deprived for several days and their sleep structure may be highly affected. While additional data may not be required to warrant the publication of this study, some rewording is necessary for the sentences "These results suggest that a reduction in sleep spindle density can activate dormant SWD" (abstract) or similarly "These results imply that a decrease in sleep spindle density can activate dormant SWDs" (l. 267). In addition, the assertion that "a decrease in sleep spindle density can provoke SWD generation" (L 274) cannot be supported by the current data. It should be made clear that decrease in spindle density is not the only possible reason.

Along the same lines, regarding the results of ethosuximide administration: the conclusion that "these findings emphasize the profound effect of SWDs on sleep spindle density" (L 205) may overstate the data. While the simultaneous decrease in SWDs and increase of spindles is intriguing, it does not necessarily indicate a causal effect of one on the other. It suggests a relationship and shared dynamic changes in the cortico-thalamo-cortical circuits but not causality. Such claims should be moderated throughout the manuscript.

Why does the SWD detection criteria differ between the PV-ArchT mice and the GBL-treated mice? Specifically, why the behavioral arrest in GBL-treated mice was not subjected to a threshold and why a lower frequency was used?

Describe more in details describe how the behavioral arrest was identified and detected.

Last but not least, there are some issues with statistical comparisons that should be addressed (see comment below).

Minor comments

Line 37: clarify what the word "which" refers to, SWDs or spindles or the relationship.

Figure 2:

In sections B and C, it would be beneficial to display all data points (The number of animals are 5) rather than mean and SEM.

In section B: Y axis label should specify density or number per hour

Provide a rationale for selecting P120 for analysis in sections B and C

The methodology section should elaborate on the experimental protocol, particularly how awake, NREM, and REM states were distinguished during the light and dark cycles: Were the same mice placed in the freely moving rig four times during the light cycle and then returned to a cage for sleep after each session?

--

Reviewer 2

In this paper, the authors studied the temporal relationship between sleep spindles and Spike-and-Wave Discharges (SWDs). Using a transgenic mice model of absence seizure and in vivo cortical EEG recordings, they showed that a decrease in sleep spindle density precedes the onset of SWDs at different ages. Additionally, they induced a decrease in sleep spindles density in WT mice by repeated injections of caffeine. In those mice, injection of low-dose gamma-butyrolactone (GBL) induced SWDs, while it did not in caffeine-untreated mice. A major claim of the paper is that it establishes a causal link between a decrease of sleep spindle density and the onset of SWDs. However, the statistics are too weak to support the paper's conclusions, and the experimental design does not allow for establishing a causal link between spindles and SWDs. The interpretation of spindle decrease as a predictor/biomarker of absence epilepsy is severely undermined by the use of a model in which SWDs are triggered. Overall, the paper lacks clarity and suffers from over-interpretation.

- Please explain the double transgenic model early (in the Introduction) and more clearly. In the results section, explain clearly what is the difference between the "spontaneous" (Fig 1) and dox-induced (Fig 4) SWDs.

- Throughout the manuscript, clarify what type of animal was used as a control (ex: Fig 3a)

- Clarify in Table 1 and methods how the detection of SWD was performed. If visually, please specify. In Table 1, express the amplitude in SDs. Change the title from "criteria" to "features" to avoid confusion with detection criteria.

- Please explain and discuss the fact that the criteria for SWD detection are different between PV-ArchT mice and GBL-treated mice. (Fig 2-3-4: 7 to 10 Hz versus Fig 6: 4 to 6 Hz 3s).

- How do you ensure that SWDs during REM sleep are different from phasic REM, known to have higher theta power than tonic REM?

- Fig3BC, Fig4E, Fig5C, Fig6BD:What does "spindle duration / min" mean ? Is it a mistake? Should it be "Spindle duration (s)"? If not, clarify.

- Fig1C: the pie chart shows the percentage of occurrence in different states but it is not controlled by the time spent by the animal in each stage. It might simply reflect sleep architecture. Please correct or delete it.

- Fig3B: Show the data at P60, P90 and P120 for the control animals. Adapt statistics accordingly.

- Fig3 legend: specify the number of animals for each group.

- Are the P60 to P120 the same animals?

- Why does the number of data points change across the density, duration and power plots in Fig. 3B?

- S.e.ms reported on the graphs seem inconsistent (lots of variation of visually similar spread of the data). Please check the statistics.

- Fig4: What is the difference between the typical SWDs and other epileptiform discharges? Why is it interesting to look at both ? Specify.

- Describe in the methods how those other epileptiform discharges were detected.

- One of the main messages is that spindle density decreases during the week preceding SWDs onset. Please additionally show and discuss the spindle density at the week of the onset (3rd week) and after the onset (6th week). This would constitute a clearer study of the temporal sequence linking spindles and SWDs onset.

- Spindle density might be influenced by the time spent in different states (wake/REM:NREM sleep). Throughout the manuscript, controls for the effects of sleep architecture and time spent in sleep are lacking. This includes baseline conditions, effects of GBL, effects of caffeine, and sleep architecture after 4 days of caffeine, before the GBL injection.

- Regardless, because caffeine reduces sleep time in parallel with spindle density, it is impossible to know whether the most accurate predictor of SWDs development is sleep reduction of selectively spindles reduction. These different interpretations should appear in the discussion as well.

- Fig5-6: Please discuss in the text the limitations of not using saline injection controls as the effects of restraint for injections might induce changes in sleep architecture/spindles.

- Rewrite the manuscript to remove the claim of causality. Even when the term "causality" is not used, some sentences imply it and should be reframed (ex : line 268;274-275). Caffeine may have other effects than decreasing the spindle density influencing low GBL-induced SWD onset. The SWD onset may be due to the caffeine-GBL interaction (discussed l. 361-364).

- Line 59-61 "This model has the advantage...". It is unclear what the advantage is.

- As specified in the text (line 61-62) "The TRN causes SWDs and also has a known role in spindle formation" : Thus, in this model, the formation of SWDs and the decrease in spindles could occur independently. Although the decrease in spindles occur before the onset of SWDs, SWDs appearance could be due to another, unidentified and longer process unrelated to spindles.

- A suggestion to more strongly back up the spindle-SWDs link is to compute the correlation between the change in spindle density (before/after caffeine) and the number of SWDs/h (the "severeness" of the phenotype) after low GBL induction. This analysis requires to increase the number of animals in the study (see point 1).

- Please show how the spindle density is affected by the low GBL (in Fig 6E; as you do in Fig5C for high GBL).

- Why does caffeine-lowGBL induce a higher number of SWDs/h than a high dose of GBL? Please discuss.

- Clarify what you mean by "dormant SWDs" (Line 29-30; 268)

- Both the introduction and discussion lack details and discussion about the existing literature relative to the link between spindles and SWD density and pharmacological studies. Examples include i) line 43-49: previous studies got interested in spindles density and SWDs. Your manuscript would be enriched by confronting your results with this already published data to better highlight what is new. ii) line 351-358: mention the main pharmacological studies earlier in the paper, as the rationale of your study builds on this. iii)line357: "Furthermore, clonidine, which increased the number of SWDs, was associated with decreasing sleep spindles". Reference the paper and develop the discussion of it since it overlaps with your study.

Other points

- Missing legend in Fig 1b for the color of the traces.

- Check multiple typos in the manuscript and figure legends and axes.

- Line 260-261: the referred figure (Fig6C) doesn't reflect the text.

- There are multiple occurrences when the text references the wrong figure, or a figure that does not show the corresponding result. (ex : l161-162 fig 1B; l164-166; referred fig 1c unrelated or not quantified)

- In Fig1C the spectrograms, the pie chart and text call the supposedly same state 3 different ways, respectively "NREM","Light sleep" and "transition time between awake to NREM sleep". Correct and complete with the appropriate state.
